# Respiratory metabolism and energetics of *Polistes* paper wasp larvae and pupae from differing climates

**DOI:** 10.1007/s00040-025-01053-x

**Published:** 2025-07-27

**Authors:** H. Kovac, A. B. Amstrup, H. Käfer, J. G. Sørensen, A. Stabentheiner

**Affiliations:** 1 Institute of Biology, University of Graz, Universitätsplatz 2, 8010 Graz, Austria; 2 Department of Biology, Aarhus University, Aarhus, Denmark

**Keywords:** Respiratory metabolism, Energetics, Paper wasp, Larva, Pupa, Climate

## Abstract

In ectotherms metabolic rate usually increases disproportionately with ambient temperature within the viable thermal range. This leads to an expectation of a physiological adaptation which balances the benefits and costs of the environmental conditions of the habitat. *Polistes* paper wasps is a globally distributed genus that inhabits a wide range of thermal environments. Their metabolism and energy requirements are expected to be dependent on life history and the environmental conditions of their habitat. We examined the CO_2_ production of larvae and pupae of three species (*P. dominula*, *P. gallicus*, *P. biglumis*) from different climates (Temperate, Mediterranean, Alpine). We investigated larvae and pupae across a temperature range of 5–45 °C. In combination with microclimate measurements, we estimated the energy requirements for an entire breeding season. The metabolic rate increased sigmoidally with increasing temperature in all species and stages. A comparison of the mass-specific metabolic rate revealed a similar relationship in all species and stages, with the exception of *P. gallicus* larvae which had a significantly lower metabolism, particularly at higher temperatures. The energy expenditure was lowest in *P. biglumis*, which was mainly attributed to the lower environmental temperatures. When calculating energy requirements under future conditions with increasing temperatures, an additional energetic demand of between 6 and 23% was observed.

**Supplementary Information:**

The online version contains supplementary material available at 10.1007/s00040-025-01053-x.

## Introduction

Energy demand is a crucial parameter of life-history traits in all animals, including insects (e.g. Chown and Terblanche 2007; Desforges et al. 2019). Metabolism provides an organism with energy from food and enables fitness- and non-fitness-related activities. Measuring metabolic rate is one way to estimate the energetic costs associated with different life functions such as growth, development, and movement among many others (Shah et al. 2021). In (ectothermic) insects, temperature is one of the most important factors determining their metabolic rate (see e.g. Chown and Gaston 1999; Chown and Nicolson 2004; Terblanche et al. 2005). Therefore, estimating the temperature dependence of metabolic rates helps to determine the cost of living and performance in a particular thermal environment. Because of the thermal sensitivity of metabolic rate (and other traits), temperature is a deciding factor in geographical distribution and abundance (Angilletta 2009; Shah et al. 2021) and can drive genetic adaptation.

Paper wasps of the genus *Polistes* are primitively eusocial wasps. They build a single, unprotected comb of highly variable size, ranging from a few to several hundred cells (see e.g. West-Eberhard 1969; Akre 1982; Reeve 1991; Höcherl and Tautz 2015; Stabentheiner et al. 2022). The brood is reared in cells in the comb, where their ability to move is restricted and they rely on food provided by the adults. They are very successful in their lifestyle and have a wide distribution range and high abundance in Europe. Although European species originate from the Mediterranean climate around the Mediterranean Sea, they have spread and are now present in almost all climatic regions of Europe (Blüthgen 1956a, b; Carpenter 1996; Pekkarinen and Gustafsson 1999; Rathjen 1999). Paper wasps can be found in natural landscapes as well as in suitable urban areas, but nesting behaviour varies between species and depends on the availability of local structures suitable for nesting and on microclimatic conditions (Pérez-Bote and Mora-Rubio 2020; Pérez-Bote et al. 2020; Stabentheiner et al. 2022).

We chose three closely related species of paper wasps (Schmid-Egger et al. 2017) from different climates for our study: *Polistes dominula* from the temperate central European climate, *Polistes gallicus* from the southern Mediterranean climate, and *Polistes biglumis* from an Alpine climate. *P. dominula* has a distribution range characterized by relatively high climatic variability with an original range spanning the Mediterranean region and the North African and Middle Eastern countries, including parts of Russia and China, and recent expansions into Northern Europe, the Americas, and Oceania (Carpenter 1996; Judd and Carpenter 1996; Cervo et al. 2000). The distribution range of *P. gallicus* is characterized by lower climatic variability in the warm, dry regions of Southern Europe and North-western Africa (Schmid-Egger et al. 2017). *Polistes biglumis* is a boreo-montane species which mainly inhabits mountainous areas with harsher climate conditions and a lower climatic variability in Europe and central Asia (Lorenzi and Turillazzi 1986; Schmid-Egger et al. 2017). All three species apply thermoregulatory measures to avoid critical high nest temperatures (Steiner 1930; Höcherl et al. 2016; Stabentheiner et al. 2022; Kovac et al. 2023). The metabolic rate of the adults of these species increases exponentially with ambient temperature and differs between species (Käfer et al. 2015; Kovac et al. 2017, 2020). However, the metabolic rates of larvae and pupae are still not known.

Larvae and pupae are very different life stages of insects which leads to different metabolic demands. Larvae typically require a significant amount of energy to support rapid growth and development. In larvae as well in pupae the metabolic rate can vary across the development stages due to the different physiological processes they undergo. A high level of activity and growth can lead to a high metabolic rate in larvae compared to other developmental stages (see e.g. Melampy and Willis 1939; Schneiderman and Williams 1953; Merkey et al. 2011; Bawa et al. 2021; Gao et al. 2022; Medina-Báez et al. 2023; Powers et al. 2024). Metamorphosis from larva to pupa is one of the key steps in the life history of holometabolous insects (see e.g. Truman and Riddiford 1999; Merkey et al. 2011; Bellés 2019; Rolff et al. 2019). Pupae do not feed but instead use their stored energy reserves to fuel their metamorphosis into adults. During this transitional stage, many physiological changes occur, including changes in gene expression patterns and the activation of processes such as autophagy and apoptosis. Also, many physiological processes are slowed or temporarily halted (Sinclair et al. 2016).

This study aimed to determine whether paper wasps from different climates exhibit a different metabolic performance. To do this, we measured the metabolic rate of the larvae and pupae of three species of paper wasps (*P. dominula*, *P. gallicus*, and *P. biglumis*) at a temperature range they likely are exposed to in their habitat during a nesting season. According to the metabolic cold adaptation hypothesis, we predicted that species from colder, more northern climates would have higher metabolic rates at a given temperature than those from warmer, more southern climates. Further, the sensitivity to temperature increase of species from cooler climates would be higher than that of species from warmer climates (e.g. Scholander et al. 1953; Nielsen et al. 1999; Addo-Bediako et al. 2002; Lardies et al. 2004; Kovac et al. 2020). The main goal of these measurements was to calculate the energy requirement of individual larvae and pupae. This was accomplished by using nest microclimate temperature records in addition to metabolic rate data. We calculated the energy requirement of individual larvae or pupae for an entire breeding season. Since paper wasp brood is strongly subject to environmental variation (Höcherl et al. 2016; Stabentheiner et al. 2022; Kovac et al. 2023), we also calculated energy requirements under scenarios of increased temperatures due to climate change. This demonstrates which species could be most strongly affected by climate change, and how metabolic demands can contribute to these wasps’ vulnerability to climate change.

## Material and methods

### Study species and origin of samples

Experiments were carried out between 2021 and 2023 on three species of paper wasps (*Polistes* ssp.) from three different climatic regions. We collected nests at two different locations for each climate region: *P. dominula* from temperate Styria, Austria (AT; Goritz, Gschwendt); *P. gallicus* from Mediterranean Tuscany, Italy (IT; Sesto Fiorentino, Trespiano) and *P. biglumis* from alpine Styria, Austria (AT; Teichalm, Heilbrunn). Ten nests of each species were collected over the three years. Specimen collection information is detailed in Supplementary table “General information”, Supplementary File S1. Nests in Austria were collected in the vicinity of the laboratory in Gschwendt (Styria, Austria), arriving at the laboratory within one hour of collection. Nests collected in Italy arrived at the laboratory approx. 24 h after collection due to the longer transport distance. We collected whole nests in the post emergence stage with larvae and pupae (but without adults). We determined the species by means of the adults on the nests. As most of the experiments were carried out in June and July, we assumed that the individuals studied (larvae and pupae) were workers and not gynes, but we did not determine their sex. Nests were stored at room temperature (~ 20 °C) until experiments. To account for differences in transport time from the collection sites to the laboratory, measurement of larvae and pupae collected in Austria was started the day after collection, whereas measurement of animals collected in Italy was started immediately on arrival at the laboratory. All experiments were completed within three days of collection.

### Measurement procedure and experimental setup

We measured CO_2_ emissions from individual larvae and pupae, which is commonly used as an indirect measure of an organism's metabolic rate. We defined our measurements as routine metabolic rate (RMR) to account for absorptive processes and locomotor activity of the larvae during respirometry trials. However, regular observation indicated that individuals were minimally active during experiments. Experiments were assayed at the ecologically relevant temperature range (5–45 °C) of the species. Eight respirometry measurement chambers were connected to an eight-channel multiplexer (RM Gas Flow Multiplexer, Sable Systems, Las Vegas, Nevada, USA) and operated in a stop-flow measurement configuration. The multiplexer controlled the sequential flushing and shut-off of the metabolic chambers, allowing the simultaneous measurement of eight individuals. During the flushing phase, the metabolic chamber was perfused with humidified air (60% rH) at a fixed flow rate of 144 ml min^−1^. After the flushing phase, the metabolic chamber was closed. The duration of the flushing phase of a chamber was 3 min, the closed phase was 21 min (7 chambers times 3 min), so an entire cycle was 28 min. The multiplexer was connected to a differential infrared carbon dioxide gas analyser (DIRGA; URAS 14, ABB, Zürich, Switzerland), which measured the CO_2_ release of the insects with an accuracy of ~ 2 ppm. To maximise the sensitivity of the system (< 0.2 ppm), the air was sampled from outside the laboratory. Before entering the reference tube of the DIRGA, the air was passed through a 10 l container to damp fluctuations in CO_2_ content, through the pump and mass flow controllers (0–1000 ml min^−1^, Brooks 5850 S), and then through another container (5 l) for additional CO_2_ and pressure fluctuation damping. To maintain a relative humidity of approximately 60% in the measurement chambers to avoid desiccation of the larvae and pupae, the air was humidified by passing it through two bottles of distilled water (Stabentheiner et al. 2012). After passing the measurement chamber the air was dried using Peltier-driven cooling traps (10 °C) before entering the URAS reference and measurement tubes (where it was heated to 60 °C). The volumes (nl) of CO_2_ production reported in this paper refer to standard (STPS) conditions (0 °C, 101.32 kPa = 760 Torr). CO_2_ release was recorded at one second intervals. At the beginning and end of each experimental run, the gas analyser was automatically calibrated to zero and an end point, using internal calibration cuvettes, and the data were corrected for any remaining drift or offset. Possible small “switching artefacts” in the CO_2_ readout caused by the multiplexer, which were highly reproducible, have been corrected according to a control trial after the experiments. In further steps of the analysis, the metabolic rate of the larvae and pupae was calculated by integrating the CO_2_ production peaks of three measurement intervals and averaging the amount of CO_2_. Metabolic rates (V̇CO_2_) are reported as both individual (non-mass-specific) V̇CO_2_ (µl min^−1^) and mass-specific V̇CO_2_ (µl min^−1^ g^−1^). As the larvae and pupae differed significantly in mass (Fig. S2), the mass-specific CO_2_ production rate (V̇CO_2_, µl min^−1^ g^−1^) was preferred.

Prior to the experiments, the nests were dissected and the larvae and pupae were carefully removed from the cells. The larvae and pupae were weighed to an accuracy of 0.1 mg (Shimadzu AUW-120DV balance, Nishinokyo Kuwabaracho, Nakagyoku, Kyoto, Japan). We used at least 13 individuals per life stage (larvae and pupae) from each species (see Table S1 for details). In terms of mass, we randomly sampled larvae of different sizes. However, we did not use larvae smaller than 20 mg (due to the limitation of the measuring system and to allow a high accuracy of CO_2_ measurement even at low temperatures). For pupae, we used a similar number of light and dark pigmented individuals (early and late-stage pupae, respectively). Specimen mass and stage information is detailed in Supplementary table “General information”, Supplementary File S1. Individual larvae or pupae were placed in small plastic tubes (Supplementary File S2, Fig. S1, inner length 20 mm, inner diameter 10 mm, volume 1.57 ml) that functioned as respiratory measurement chambers. Eight of these chambers were arranged in parallel and placed in a water bath (Julabo F33 HT, JULABO Labortechnik GmbH, Seelbach, Germany) to regulate the temperature with an accuracy of ± 0.1 °C during the experiments. The experiments were performed in a temperature range from 5 to 45 °C in 10 °C increments. For measurements in the temperature range of 15–45 °C the following procedure was used. The wasps were put in the water bath and after 45 min of habituation at 15 °C, the experiment began. The wasps remained at this temperature (15 °C) for 90 min and then the temperature was increased by 10 °C within 15 min. Again, after 45 min of habituation at the increased test temperature, the measurement started. This procedure was repeated until the highest test temperature was reached and measurement was completed. The whole experiment took about six and a half hours. The experiments at the test temperature of 5 °C were carried out in the same way, but in an independent experiment with only this target temperature (due to technical constraints with measurements at this low temperature).

To assess the thermal sensitivity of the metabolic rate, we determined the Q10. Q10 is a measure of the change in metabolic rate with a 10 °C change in temperature. We calculated the change in metabolic rate over four temperature intervals (5–45 °C) as Q10 values. Averaging the three Q10 values of the exponential part of the fitted curves (5–35 °C), provided a metric for the acute thermal sensitivity of the metabolic rate.

### Respiratory quotient

Additional experiments were carried out to determine the respiratory quotient (RQ) for energetic calculations of the wasp’s energy turnover during the breeding season. The CO_2_ measurement device was the same as described above (DIRGA), with the addition of an oxygen analyser (Oxzilla 2 differential oxygen analyser; Sable Systems International, Las Vegas, USA). The measurement procedure was similar, but only at one test temperature (25 °C), with 9–17 individuals of each stage per species (see Table S4). Eight individuals were placed individually in the respirometry chambers described above and remained there for approximately 100 min. In these experiments, commercial dried air was supplied to the reference and measurement channels (in parallel mode) of a serial arrangement of the DIRGA and Oxzilla devices. The multiplexer switched the measurement channels between the 8 chambers in sequential order at 4 min intervals (Supplementary File S2, Fig. S7). After leaving the measurement chambers with the larvae or pupae, the air passed through a desiccant (Drierite; W. A. Hammond Drierite Co. Ltd, Xenia, OH, USA) before entering the system (DIRGA and Oxzilla). The difference between the measurement and reference channels was used to compensate for any instrument drift and offset during evaluation. Data acquisition was performed using the DIRGA CO_2_ gas analyser system software (Centrol 5, Harnisch, Graz, Austria). After drift and offset correction, the accumulated CO_2_ and consumed O_2_ were calculated by integrating the signals over time. The respiratory quotient was then calculated as the quotient of the integrals (RQ = ∫CO_2_/∫O_2_). The systems were calibrated before and after the experimental runs.

### Nest microclimate measurement

We measured the microclimate at the nests in the habitat of the three species. Measurements were made throughout the wasps' breeding season (May–August) in the years 2021–2023. Additional temperature data for the years 2018–2020 were used from a previous study (Kovac et al. 2022a; Fig. S3; *P. dominula* AT: N1-N8, *P. gallicus* IT: N1-N6, *P. biglumis* AT: N1-N7), so that we had temperature data from 10 nests for each species. Nest ambient temperature was continuously recorded at 10 min intervals with data loggers (MSR Electronics GmbH, Seuzach, Switzerland; and Extech SD 200, FLIR Commercial Systems, Nashua, NH, USA) placed 1–5 cm from the nest. The temperature sensor was protected by an aluminum cover to avoid heating by direct solar radiation.

### Energetic expenditure calculation

The energy expenditure of a single larva and pupa was calculated for each species, life stage and nest. The carbon dioxide production of individuals was calculated chronologically for the 10 min intervals (the interval of the temperature recordings) and then the energy expenditure was calculated using the nest temperature data and the metabolic rate equations (Tables S2 and S3). To do this, we first converted CO_2_ production to O_2_ consumption using the respiratory quotient determined for each species and stage (see Table S4), and then multiplied the O_2_ consumption by the appropriate caloric equivalent (see Silbernagl et al. 2018). Then, the energy turnover was calculated chronologically for the 10-min intervals and summed up for the whole study period (cumulative costs from May to August). Taking into account that the nesting season starts earlier in Italy than in Austria, we calculated the costs for *P. gallicus* IT from the beginning of May until the end of July, and for the Austrian species *P. dominula* AT and *P. biglumis* AT from the middle of May until the middle of August (92 days each, respectively). As the individuals differed significantly in mass, we also calculated the costs with the mass-specific metabolic rate by using the mean mass of the investigated larvae and pupae. We are aware that the assumption presented is simplistic, as larvae grow during development and the duration of development depends on ambient temperature. Furthermore, we calculated energy expenditure for the entire breeding season and not for a development period. Therefore, results present an average of energetic costs for an “average” larva or pupa over the entire breeding season.

We simulated the additional energetic costs for larvae and pupae for a breeding season with temperature increases due to climate change. We did this in a simple way by adding 1, 2 and 3 °C to the actual recorded nest temperature and calculating the energetic costs with the elevated temperature.

### Data analysis and statistics

All calculations were performed using MS Excel (Microsoft Corporation, Redmond, WA, USA). Curve fitting was performed using Origin 2017 software (OriginLab, OriginLab Corporation, Northampton, MA, USA) which provides several sigmoid fitting functions for the analysis of metabolic rate data. The fit was optimised by iteratively varying the coefficients according to the nonlinear least squares Levenberg–Marquardt (L–M) algorithm, an iterative procedure that combines the Gauss–Newton method and the steepest descent method (Origin Help 2022; see also Stabentheiner and Kovac 2023). Associated statistics was performed using Statgraphics software (Statgraphics Centurion XVI, StatPoint Technology Inc., The Plains, VA, USA). Non-parametric Kruskal–Wallis tests were used to compare individual mass and cumulative energy expenditure, and the Bonferroni test was used for pairwise comparisons of these data. To test the influence of the independent variables (ambient temperature, nest and species/life stage affiliation) on the dependent variable (metabolic rate), we first applied a multifactorial ANOVA. To perform the ANOVAs, we did a log10 transformation on the exponential part of the V̇CO_2_ of the model fits (5–35 °C). We excluded the V̇CO_2_ data at 45 °C because they were already in a critical temperature range for respiration (CT_max_, see Käfer et al. 2025). To compare metabolic data (log-transformed linear fits) from different life stages or species, an additional ANOVA was performed to test for differences in the intercept or slope of the fitted curves. To account for different masses of individuals, we performed a multifactorial ANOVA with temperature and species as main effects and mass as a covariate. All detailed statistics can be found in Supplementary Files S1 and S2.

## Results

### Metabolic rate

**Fig. 1:**
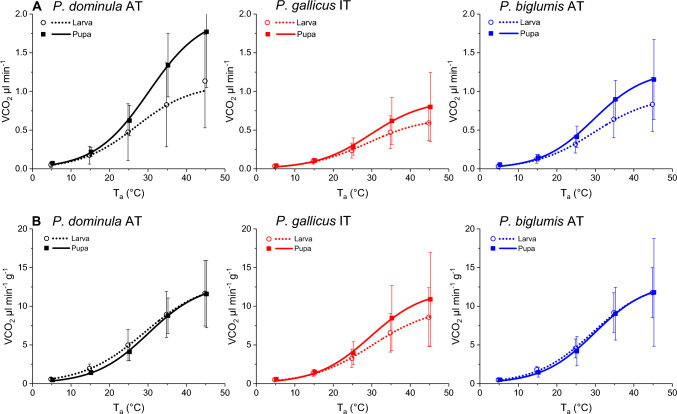
Individual **A** and mass-specific **B** metabolic rate of paper wasp larvae and pupae from Austria (*P. dominula* AT, *P. biglumis* AT) and Italy (*P. gallicus* IT) in relation to ambient temperature (T_a_). Symbols represent means and error bars the standard deviation

Metabolic rate (CO_2_ production) increased sigmoidally with temperature in all species and stages (Fig. 1; Supplementary File S2, Table S1, S2, and S3). In terms of individual (non mass-specific) metabolic rate, pupae always showed a higher metabolic rate than larvae (Fig. 1A). To account for mass variation, we also calculated the mass-specific metabolic rate. Here we found that the metabolic rate was very similar in all species and stages with only one exception, the larvae of *P. gallicus* IT (Fig. 1B). The mean mass-specific metabolic rate (V̇CO_2_) ranged from 0.35 to 11.8 µl min^−1^ g^−1^ (5 to 45 °C). As the larvae and pupae differed significantly in mass (except *P. gallicus* IT, Supplementary File S2, Fig. S2), we will focus on the mass-specific metabolic rate. In *P. dominula* AT and *P. biglumis* AT the metabolic rate of larvae was slightly higher than that of pupae at lower temperatures, but the difference was not statistically significant (*p* > 0.05, ANOVA; Supplementary File S2, Table S5). However, in *P. gallicus* IT, larvae had a significantly lower metabolic rate than pupae at higher ambient temperatures, i.e. their thermal sensitivity was lower (Fig. 1; *p* < 0.05, ANOVA; Supplementary File S2, Table S5). The interspecific comparison of the mass-specific metabolic rate showed significantly lower values for *P. gallicus* IT larvae compared to the other two species (*p* < 0.01, ANOVA; Supplementary File S2, Fig. S4, Table S6). In pupae differed only *P. dominula* AT from *P. gallicus* IT (*p* < 0.05, ANOVA; Supplementary File S2, Fig. S4, Table S6). An analysis with nest id as random factor revealed differences for nests in all species (*p* < 0.01, ANOVA; Supplementary File S2, Table S8).

The temperature sensitivity of the metabolic rate (Q10) for the exponential part of the curve (5–35 °C; Table 1) was very similar in the three species and did not differ significantly (larvae: *p* = 0.8602, H = 0.3012, df = 2; pupae: *p* = 0.9017, H = 0.2070, df = 2; Kruskal–Wallis test). In larvae the mean Q10 was in the range of 2.2 to 2.4 and in pupae it was 2.6. Due to the sigmoid (logistic) nature of the whole relationship it decreased with increasing temperature and was not different between pupae and larvae (*p* = 0.2318, U = 54.5, N = 9; Mann–Whitney test).

**Table 1:** Temperature sensitivity of metabolic rate of paper wasp larvae and pupae from Austria (*P. dominula* AT, P. *biglumis* AT) and Italy (*P. gallicus* IT). Change in metabolic rate for every 10 °C increase in temperature (Q10; 5–45 °C). Kruskal -Wallis-test revealed no effect of species on Q10 (5–35 °C; larva *p* = 0.8068, pupa *p* = 0.7872)

Species	Developmental stage	5–15 °C	15–25 °C	25–35 °C	35–45 °C	Mean (5–35 °C)
*P. dominula* AT	Larvae	3.5	2.7	1.7	1.2	2.7
	Pupae	3.8	3.1	2.1	1.3	3.0
*P. gallicus* IT	Larvae	3.2	2.7	1.9	1.3	2.6
	Pupae	3.8	3.1	2.0	1.3	3.0
*P. biglumis* AT	Larvae	3.6	2.9	1.9	1.3	2.8
	Pupae	4.0	3.2	2.1	1.3	3.1

### Respiratory quotient

The mean respiratory quotient of larvae and pupae was similar in all species. It was in the range of 0.80 to 0.88 with only one exception, the larvae of *P. biglumis* AT had a respiratory quotient of 1.02 (Table S4).

### Nest microclimate and energetic expenditure

The three locations differed significantly in their microclimate (*p* < 0.0001, H = 41214.3, df = 2, Kruskal–Wallis-test). Mean ambient nest temperature during the breeding season was highest in Mediterranean Italy (24.8 °C), slightly lower in temperate Austria (23.7 °C) and lowest in Alpine Austria (18.9 °C). The local climate values of the breeding season (mean of nearest weather stations; May–August 1981–2010) were considerably more different and lower, amounting to 22.9 °C, 18.0 °C and 13.0 °C, respectively (LaMMA Consorzio, 2021; ZAMG - Zentralanstalt für Meteorologie und Geodynamik, 2021). The climate normal values (1981–2010), which characterize the climate of the study areas, were even lower, amounting to 15.5 °C, 11.1 °C and 5.2 °C, respectively (Supplementary File S1, Climate data; Supplementary File S2, Fig. S3).

**Fig. 2:**
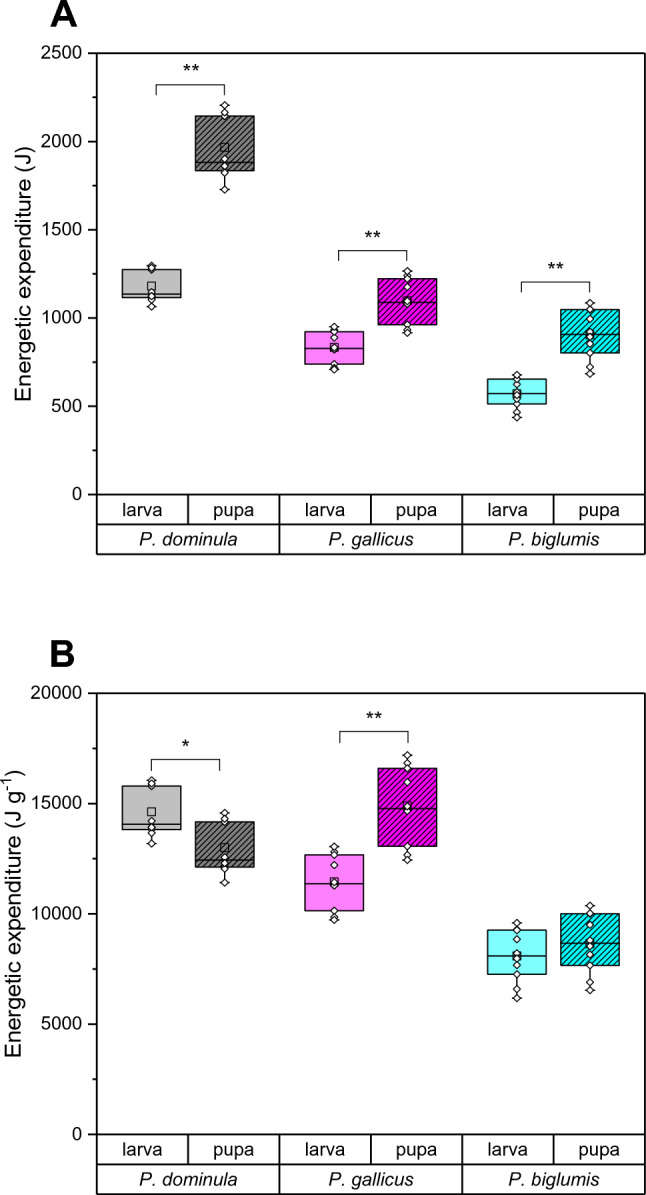
Individual **A** and mass-specific **B** cumulative energetic expenditure of paper wasp larvae and pupae from Austria (*P. dominula* AT, *P. biglumis* AT) and Italy (*P. gallicus* IT), calculated with metabolic rate data and microclimate data for a breeding season from May to August. Climate data were recorded at six years (2018–2023), different breeding seasons were used for different species. Box and whisker plots represent median mass with first and third quartiles; dots in plots are means (* significant difference *p* < 0.05, ** significant difference *p* < 0.01; Mann–Whitney test)

We calculated the energetic expenditure for each nest with the ambient nest temperature and the metabolic fit functions and summed them up for the entire breeding season (Supplementary File S2, Fig. S5 and S6). The mean individual energy expenditure (Fig. 2A) at the end of the season was in the three species always higher in pupae than in larvae (*P. dominula* AT: *p* < 0.0002, U = 100.0, N = 10; *P. gallicus* IT: *p* < 0.0008, U = 95.0, N = 10; *P. biglumis* AT: *p* < 0.0003, U = 99.0, N = 10; Mann–Whitney test). This was mainly because pupae were heavier, but in *P. gallicus* IT also because of the lower metabolic rate of the larvae. When looking at the mass-specific energy expenditure, the results were more diverse (*P. dominula, P. gallicus, P. biglumis* larvae: 14628, 11390, 8339 J g^−1^; pupae: 13001, 14703, 8635 J g^−1^; Fig. 2B). While in *P. dominula* AT the larvae had a higher mass-specific energy expenditure (*p* = 0.03749, U = 22.0, N = 10; Mann–Whitney test), in *P. gallicus* IT the pupae had a higher expenditure (*p* = 0.00132, U = 93.0, N = 10). In *P. biglumis* AT there was no difference (*p* = 0.52052, U = 59.0, N = 10). Interspecific comparison of larval energy expenditure revealed significant differences between species (*p* < 0.0001, H = 25.3063, df = 2, Kruskal–Wallis test), with each species being significantly different from the other (*p* < 0.05, Bonferroni test). In the pupae there was also a significant difference between the species (*p* < 0.0001, H = 22.0023, df = 2), with the pairwise comparison showing differences in *P. biglumis* AT pupae with the other two species (*p* < 0.05, Bonferroni test), but not between *P. dominula* AT and *P. gallicus* IT pupae. Overall, *P. biglumis* AT had the lowest energy expenditure for a breeding season (Fig. 2B).

**Fig. 3:**
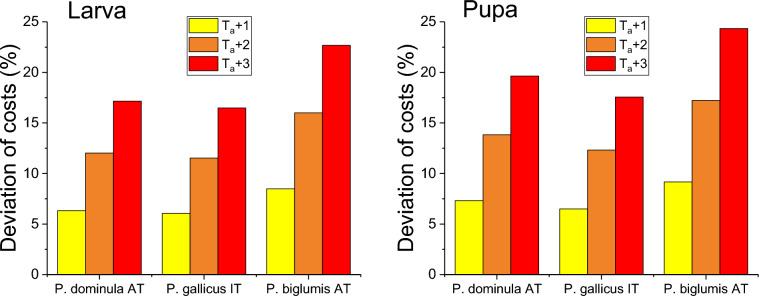
Percent deviation in energetic costs of paper wasp larvae and pupae from Austria (*P. dominula* AT, *P. biglumis* AT) and Italy (*P. gallicus* IT) in future climate scenarios, with a temperature increase of 1, 2, and 3 °C above ambient air temperature in comparison to recorded microclimate measurements at the nests during a breeding season from May to August

The simulation of an increased ambient temperature (Fig. 3) resulted in an almost linear increase in energetic costs with temperature. The calculated additional costs (relative costs) ranged from 6.1% to 22.7% for larvae and from 6.5% to 24.3% for pupae. The highest additional costs were observed for the alpine *P. biglumis* AT.

## Discussion

Individual and mass-specific V̇CO_2_ exhibited a strong correlation with temperature for both larvae and pupae. However, logistic (sigmoid) curves fitted the relationships between metabolic rate and ambient temperature (T_a_) better than exponential curves in both life stages (Fig. 1). Logistic relationships indicate that at high temperatures (> ~ 35 °C) destructive metabolic effects become more and more important (Willmer et al. 2004). This coincides with the finding that up to 35 °C no increased induction of metabolic repair mechanisms, i.e. the expression of heat shock proteins like Hsp70, Hsp83 and Hsc70, was measured (Amstrup et al. 2024).

Surprisingly, the change of the mass-specific metabolic rate with temperature of larvae and pupae was quite similar, or even lower in larvae of *P. gallicus* IT (Fig. 1). We had expected larvae to exhibit the highest metabolic rates, associated with rapid growth, and pupae to exhibit lower metabolic rates as pupal stages are not so energy demanding. In honeybees, for example, maximum oxygen consumption and carbon dioxide production occur during early larval life, when there is rapid growth. With the onset of pupation, the metabolic rate is reduced to a minimum, followed by a slight increase before emergence (Melampy and Willis 1939). In *Helicoverpa armigera*, a Lepitoptera species, Jiang et al. (2019) reported a transient increase in metabolism during the larval-larval molt and larval-pupal transition, followed by a sharp decrease during the pupal stage and a subsequent increase before eclosion. In *Helicoverpa punctigera*, the mean mass-specific CO_2_ emission of larvae was also significantly higher than that of pupae (Bawa et al. 2021). Differences in metabolic rates between life stages can be attributed to several factors, such as ontogeny, activity, body mass, or feeding (Terblanche et al. 2005). Our results may be aberrant due to the fact that we did not measure very small larvae (< 20 mg, Fig. S2). In honeybees (Melampy and Willis 1939; Petz et al. 2004) and wax moths (Schmolz and Lamprecht 2000), a high mass-specific metabolic rate was observed only during the early development phase when the larvae were very small.

Our investigation allows also a verification of the metabolic cold adaptation hypothesis (MCA) as we investigated species from different climates. The hypothesis predicts that species from cooler climates have a higher metabolic rate than those from warmer climates (e.g. Addo-Bediako et al. 2002; Oikawa et al. 2006; Terblanche et al. 2009; Bozinovic et al. 2011; Williams et al. 2016). However, in the present study, the mass-specific metabolic rate of pupae was almost identical across all species from the different climates (Fig. 1B and Fig. S4-B, Table S6). Only the results obtained in larvae partially met the expectations of the MCA. Larvae from the Mediterranean climate (*P. gallicus* IT) exhibited a significantly lower metabolism compared to those from cooler climates (*P. dominula* AT, *P. biglumis* AT) (Fig. 1B and Fig. 4S-B, Table S6). However, there was no significant difference between the species from the temperate climate (*P. dominula* AT) and the cool alpine climate (*P. biglumis* AT). These findings show that the MCA cannot be seen as a general rule in insects. With regard to the adults of polistine paper wasps, which are most relevant to our investigation, there are also controversial results. Summer individuals (workers) of the temperate *P. dominula* and the Mediterranean *P. gallicus* exhibited a very similar metabolic performance (Kovac et al. 2020), indicating no metabolic cold adaptation. The workers of the alpine *P. biglumis* even exhibited a significantly lower metabolic performance than the temperate *P. dominula*, which contradicts the MCA (Kovac et al. 2020). In the cool, alpine climate with short seasons for development the necessity to save energy is high, because the adults are unable to fly out for food at low temperature (< ~ 18 °C). An increasing metabolic rate at low temperatures would rather hinder development than promote it, especially during longer periods of bad weather. Conversely, in overwintering paper wasp gynes of the temperate *P. dominula* and the Mediterranean *P. gallicus*, the MCA was confirmed (Kovac et al. 2022b).

The Q10 in the exponential part of the metabolic curves (5- < 40 °C), which describes the thermal sensitivity of the metabolic rate, was very similar for all species, and therefore also did not confirm the MCA. The Q10 values (Table 1) of larvae and pupae were highest at low temperatures (5 °C), but at higher temperatures they decreased and were similar to those observed by Bawa et al. (2021) in *Helicoverpa punctigera* larvae and by Powers et al. (2024) in *Lymantria dispar* larvae. Therefore, our investigation of *Polistes* larvae and pupae adds additional knowledge to the much-debated controversial results found in studies on metabolic cold adaptation in insects and other arthropods (see e.g. Clarke 1991; Addo-Bediako et al. 2000; White et al. 2012). While some studies confirm the hypothesis (Scholander et al. 1953; Chappell 1983; Sømme et al. 1989; Berrigan and Partridge 1997; Chown 1997; Terblanche et al. 2009; Bruning et al. 2013; Williams et al. 2016; Kovac et al. 2022b), others report the absence of metabolic cold adaptation (Lee and Baust 1982; Oikawa et al. 2006; Alton et al. 2016; Messamah et al. 2017) or mixed effects (Aunaas et al. 1983), or even report opposite findings (Lardies et al. 2004; Kovac et al. 2020). In general, it is still unclear to what extent it is possible for temperature to drive adaptation (Sørensen et al. 2018). The idea of thermodynamic constraint proposes that temperature defines biochemical rates which in turn determines organismal traits including the metabolic rate (at least in the standard or basal metabolic rate, i.e. the cost of organismal maintenance). The here quoted studies illustrate the complexity of the topic, and in field studies such as ours it is often challenging to discern the influence of “species”, “climate” and “adaptation or acclimation” on the insect’s physiological traits. We agree with Glazier's (2015) 'Adaptable Informed Resource Use (AIRU) model', which calls for a more comprehensive understanding of physiological processes. The model proposes multi-directional interactions between metabolic rate, body size, temperature, biological processes, and ecological factors. To achieve this, the experimental basis of theoretical considerations needs improvement.

The main purpose of this study was to determine the energy expenditure of larvae and pupae during a breeding season, which is crucial for their distribution and survival in a changing world with rising temperatures. The total energy expenditure of a breeding season reflects the unique environmental conditions of the habitats. Our main finding of the energetic calculations was that the insects' energy expenditure is primarily determined by the microclimate at the nest, because the mass-specific metabolic rate-temperature relationship was very similar in all species and developmental stages (Fig. 1). For the energetic calculations we measured the ambient temperature at the nests. Despite a larger difference in local climate values (4.9 °C) and climate normal values (4.4 °C), the mean ambient temperature at the nests in the temperate habitats of *P. dominula* in Austria and the Mediterranean habitats of *P. gallicus* in Italy only differed by 1.1 °C (Fig. S3). This is attributed to the ability of the foundress wasps in the temperate climate to find nesting sites in sheltered places with a favourable thermal environment (Kovac et al. 2017, 2023). In contrast, the harsher climate of alpine habitats resulted in conspicuously lower ambient nest temperatures in *P. biglumis* (Fig. S3), leading to the lowest energy expenditure of both larvae and pupae in mass-specific and mass-independent energy expenditure (Fig. 2). The higher individual energy expenditure in pupae than in larvae (Fig. 2A) was primarily due to the higher mass of the pupae. *P. biglumis* adults partly compensate for the lower ambient temperature by building their nests exposed to the (morning) sun (Steiner 1930; Stabentheiner et al. 2022).

A study on energetic costs of adults of the three species investigated showed similar results (Kovac et al. 2022a). Workers of the alpine *P. biglumis* had the lowest energy expenditure for resting and mixed activity during the breeding season, likely due to the harsh climate conditions that force them to adopt an energy-saving lifestyle. Model calculations (Kovac et al. 2023) as well as the impressive verification of their validity by direct measurements of energy stores before and after overwintering (Stabentheiner et al. 2024) also demonstrated the importance of microclimate for the energetics of paper wasps. They found that the energy requirements of overwintering gynes from temperate Austria (*P. dominula*), with lower hibernaculum temperatures, were lower than those from Mediterranean Italy (*P. dominula*, *P*. *gallicus*). These examples highlight the importance of microclimate recordings in natural systems to detail the abiotic conditions experienced by organisms in their microhabitat (see also Gril et al. 2023; Turnbull et al. 2023). This is especially important in ectothermic insects, as their body temperature mostly corresponds to the ambient temperature. Depending on its microhabitat, an insect may experience varying thermal profiles, which can differ greatly from large-scale climate data (Woods et al. 2015; Sheldon and Dillon 2016; Kovac et al. 2023). Reliable energetic calculations and predictive distribution models can only be achieved through the use of microclimate and microhabitat data (Pincebourde and Salle 2020; Pincebourde and Woods 2020).

Our model calculations are the first estimation of the energetic costs of development in these insects. They can be used to better understand the physiological and ecological effects of thermal changes and to create predictive models of the response to climate change. Model calculations conducted with the simple assumption of a temperature increase of 1, 2, and 3 °C, yielded interesting results. The additional energetic costs (relative costs increase) range from about 6% to 24%, with the highest increase observed in the alpine species *P. biglumis* (Fig. 3). In habitats with lower temperatures, even a slight increase in temperature can have a significant impact due to the nearly exponential nature of the metabolic rate-temperature relationship at low temperatures (i.e. in the lower end of our sigmoid metabolic curves). Kovac et al. (2022a) conducted a comparable study on adult wasps. The investigated species experienced additional energetic costs of 24.7%, 9.0%, and 20.4% for the standard metabolic rate at a temperature increase of 2 °C (*P. dominula* AT, *P. gallicus* IT, *P. biglumis* AT). The present study showed that the larvae and pupae of *P. dominula* AT, *P. gallicus* IT, and *P. biglumis* AT are expected to experience additional costs of 12.0%, 11.5%, 16.0%, and 13.8%, 12.3%, 17.2%, respectively, due to a 2 °C temperature increase. Overwintering gynes in hibernacles, by contrast, will experience considerably higher additional costs of 32.8%, 29.9%, and 26.2% for *P. dominula* AT, *P. dominula* IT, and *P. biglumis* AT, respectively (Kovac et al. 2023). The impact of the temperature increase appears to be more pronounced in adults, likely due to their higher metabolic rate. This also implies that the elevated costs associated with the necessity of self-preservation may reduce the capacity to provide the brood with an adequate food supply.

We are aware, however, that more research is necessary on how *Polistes* larvae and pupae are affected by increasing temperature, as it can influence various parameters. Many studies have demonstrated that higher temperatures accelerate development rates, and lower temperatures lengthen development (e.g. Partridge et al. 1994; Gibert and De Jong 2001; Petavy et al. 2001; Angilletta et al. 2002; Folguera et al. 2010). However, the acceleration of development is only possible if the adults provide sufficient food, assuming there is enough prey available. Higher temperatures could also pose a threat if they reach a detrimental level. Even in temperate Austria ambient temperatures of about 47 °C have already been measured at *P. dominula* nests, which is very close to the critical thermal maximum of the adults (47.4 °C; Kovac et al. 2017). At high temperatures, adult wasps must cool nest and brood by dispersing water droplets and fanning (Steiner 1930; Höcherl et al. 2016; Stabentheiner et al. 2022). Water collection reduces the time available for collecting food, and incurs additional energetic costs for the adults. As a potential survival strategy, the species could search for cooler nesting sites in their current habitats or migrate to cooler environments. Dispersion has already been observed in *P. dominula*, which has expanded its range to northern Germany and Denmark in recent times (Pekkarinen and Gustafsson 1999; Smit 2003; Woydak 2006).

Our study demonstrates the complex interaction of ecophysiological parameters and the potential impact of climate change on an ectothermic insect. The larvae and pupae of the three species studied, originating from different climates, show a very similar metabolic performance. However, different microclimates lead to differences in energy requirements during the breeding season. The increase in temperature caused by climate change increases the wasps' energy requirements. It seems to have a stronger effect on the alpine species *P. biglumis* than on the other species.

## Supplementary Information

Below is the link to the electronic supplementary material.

Supplementary file1 (XLSX 28 KB)

Supplementary file2 (DOCX 3127 KB)

## Data Availability

The data that supports the findings of this study are available in the Supporting Information of this article.
