# Supplementary material for: Respiratory metabolism and energetics of Polistes paper wasp larvae and pupae from differing climates
Source: Insectes Soc. 2025 Jul 27;73(1):145–56. doi: 10.1007/s00040-025-01053-x (PMC12901215; doi:10.1007/s00040-025-01053-x)

**Respiratory metabolism and energetics of *Polistes* paper wasp larvae and pupae from differing climates**

H. Kovac*^1^, A.B. Amstrup*^1,2^, H. Käfer^1^, J.G. Sørensen^2^, A. Stabentheiner^1^
*^1^* *Institute of Biology, University of Graz, Graz, Austria*
*^2^ Department of Biology, Aarhus University, Aarhus, Denmark*

*Corresponding authors at Institute of Biology, University of Graz, Universitätsplatz 2, 8010 Graz, Austria. E-mail addresses: [helmut.kovac@uni-graz.at](mailto:helmut.kovac@uni-graz.at) (H. Kovac), [anton.stabentheiner@uni-graz.at](mailto:anton.stabentheiner@uni-graz.at) (A. Stabentheiner)

Table S1. Individual and mass-specific metabolic rate (MR) of paper wasps’ larvae and pupae from Austria (*P. dominula* AT, P. *biglumis* AT) and Italy (*P. gallicus* IT).

| Species  stage | T_a_ | Mass (g) | Individual MR  (mean ± SD)  (µl min^-1^) | Mass-specific MR  (mean ± SD)  (µl min^-1^ g^-1^) | N | stage | Mass (g) | Individual MR  (mean ± SD)  (µl min^-1^) | Mass-specific MR  (mean ± SD)  (µl min^-1^ g^-1^) | N |
| --- | --- | --- | --- | --- | --- | --- | --- | --- | --- | --- |
| ***P. dominula* AT** |  |  |  |  |  |  |  |  |  |  |
| larva | 5 | 0.086±0.039 | 0.05±0.03 | 0.57±0.20 | 18 | pupa | 0.152±0.009 | 0.07±0.02 | 0.48±0.10 | 17 |
|  | 15 | 0.088±0.046 | 0.18±0.11 | 1.96±0.57 | 17 |  | 0.152±0.023 | 0.22±0.06 | 1.44±0.29 | 23 |
|  | 25 | 0.088±0.046 | 0.47±0.37 | 4.98±2.02 | 17 |  | 0.152±0.023 | 0.62±0.20 | 4.11±1.16 | 23 |
|  | 35 | 0.088±0.046 | 0.83±0.54 | 8.94±2.96 | 17 |  | 0.152±0.023 | 1.34±0.41 | 8.78±2.28 | 23 |
|  | 45 | 0.088±0.046 | 1.13±0.60 | 11.68±4.24 | 17 |  | 0.152±0.023 | 1.77±0.72 | 11.58±4.34 | 23 |
| ***P. gallicus* IT** |  |  |  |  |  |  |  |  |  |  |
| larva | 5 | 0.073±0.016 | 0.04±0.02 | 0.51±0.19 | 18 | pupa | 0.073±0.014 | 0.04±0.01 | 0.58±0.14 | 17 |
|  | 15 | 0.073±0.016 | 0.10±0.04 | 1.36±0.42 | 22 |  | 0.074±0.012 | 0.11±0.03 | 1.47±0.38 | 22 |
|  | 25 | 0.073±0.016 | 0.24±0.10 | 3.27±1.19 | 22 |  | 0.074±0.012 | 0.29±0.11 | 3.92±1.49 | 22 |
|  | 35 | 0.073±0.016 | 0.47±0.21 | 6.57±2.49 | 22 |  | 0.074±0.012 | 0.62±0.30 | 8.47±4.21 | 22 |
|  | 45 | 0.073±0.016 | 0.59±0.23 | 8.58±3.81 | 22 |  | 0.074±0.012 | 0.80±0.45 | 10.89±6.05 | 21 |
| ***P. biglumis* AT** |  |  |  |  |  |  |  |  |  |  |
| larva | 5 | 0.071±0.030 | 0.03±0.02 | 0.48±0.16 | 17 | pupa | 0.107±0.016 | 0.05±0.02 | 0.51±0.11 | 13 |
|  | 15 | 0.071±0.022 | 0.13±0.06 | 1.81±0.44 | 20 |  | 0.104±0.020 | 0.14±0.04 | 1.46±0.61 | 18 |
|  | 25 | 0.071±0.022 | 0.32±0.11 | 4.55±1.23 | 20 |  | 0.104±0.020 | 0.41±0.14 | 4.20±1.90 | 18 |
|  | 35 | 0.071±0.022 | 0.64±0.24 | 9.18±2.57 | 20 |  | 0.104±0.020 | 0.90±0.24 | 9.04±3.40 | 18 |
|  | 45 | 0.071±0.022 | 0.83±0.35 | 11.76±3.22 | 20 |  | 0.104±0.020 | 1.15±0.52 | 11.78±6.96 | 18 |

Table S2**.** Statistical details and fit parameters of sigmoid fit functions (V̇CO_2_ = a/(1 + exp(-k*(x-xc)))) of the individual metabolic rate (V̇CO_2_ in µl min^-1^) of paper wasps larvae and pupae from Austria (*P. dominula* AT, P. *biglumis* AT) and Italy (*P. gallicus* IT).

| Species | Larva | Parameter |  |  | R^2^ | P | N |
| --- | --- | --- | --- | --- | --- | --- | --- |
|  | Pupa | a | xc | k |  |  |  |
| ***P. dominula* AT** | L | 1.09224 | 26.88197 | 0.13901 | 0.43204 | <0.0001 | 86 |
|  | P | 1.99162 | 30.19853 | 0.14246 | 0.73505 | <0.0001 | 109 |
| ***P. gallicus*** **IT** | L | 0.66839 | 28.93533 | 0.13179 | 0.66909 | <0.0001 | 106 |
|  | P | 0.89444 | 29.72675 | 0.14275 | 0.57285 | <0.0001 | 103 |
| ***P. biglumis* AT** | L | 0.94523 | 29.68819 | 0.13268 | 0.69575 | <0.0001 | 97 |
|  | P | 1.27546 | 29.51716 | 0.14866 | 0.71300 | <0.0001 | 85 |

Table S3**.** Statistical details and fit parameters of sigmoid fit functions (V̇CO_2_ = a/(1 + exp(-k*(x-xc)))) of the mass-specific metabolic rate (V̇CO_2_ in µl g^-1^ min^-1^) of paper wasps larvae and pupae from Austria (*P. dominula* AT, P. *biglumis* AT) and Italy (*P. gallicus* IT).

| Species | Larva | Parameter |  |  | R^2^ | P | N |
| --- | --- | --- | --- | --- | --- | --- | --- |
|  | Pupa | a | xc | k |  |  |  |
| ***P. dominula* AT** | L | 13.28843 | 29.16155 | 0.12465 | 0.74498 | <0.0001 | 86 |
|  | P | 12.98909 | 30.07496 | 0.14306 | 0.77193 | <0.0001 | 109 |
| ***P. gallicus*** **IT** | L | 9.938950 | 30.10670 | 0.12641 | 0.67062 | <0.0001 | 106 |
|  | P | 12.17870 | 29.65938 | 0.14321 | 0.57237 | <0.0001 | 103 |
| ***P. biglumis* AT** | L | 13.18496 | 29.25255 | 0.13647 | 0.82822 | <0.0001 | 97 |
|  | P | 13.11714 | 29.81250 | 0.14590 | 0.58209 | <0.0001 | 85 |

Table S4**.** Respiratory quotient (RQ) of paper wasps’ larvae and pupae from Austria (*P. dominula* AT, P. *biglumis* AT) and Italy (*P. gallicus* IT).

| Species | Developmental | RQ | SD | max | min | n |
| --- | --- | --- | --- | --- | --- | --- |
|  | stage |  |  |  |  |  |
| ***P. dominula* AT** | Larvae | 0.88 | 0.13 | 1.26 | 0.62 | 17 |
|  | Pupae | 0.86 | 0.08 | 1.06 | 0.63 | 14 |
| ***P. gallicus*** **IT** | Larvae | 0.86 | 0.16 | 1.37 | 0.58 | 12 |
|  | Pupae | 0.80 | 0.09 | 0.98 | 0.60 | 12 |
| ***P. biglumis* AT** | Larvae | 1.02 | 0.16 | 1.42 | 0.73 | 9 |
|  | Pupae | 0.85 | 0.09 | 1.01 | 0.65 | 12 |

Table S5. Intraspecific comparison of mass-specific metabolic rate of larvae vs. pupae. ANOVA was used to test for effects of temperature and life stage on metabolic rate. Further ANOVAs test for differences in intercept and slope in linear fits of life stages.

***P. dominula* AT** Analysis of Variance

| Source | Sum of Squares | Df | Mean Square | F-Ratio | P-value |
| --- | --- | --- | --- | --- | --- |
| Covariates |  |  |  |  |  |
| T_a_ | 31.3315 |  | 31.3315 | 1428.02 | 0.0 |
| Main effects |  |  |  |  |  |
| Model | 0.11286 | 3 | 0.11286 | 5.14 | 0.0247 |
| Residuals | 3.33496 | 152 | 0.0219405 |  |  |
| Total (corr.) | 34.6668 | 154 |  |  |  |

Further ANOVA: Larvae vs. pupae

| Source | Sum of Squares | Df | Mean Square | F-Ratio | P-value |
| --- | --- | --- | --- | --- | --- |
| T_a_ | 31.219 | 1 | 31.219 | 1421.21 | 0.0 |
| Intercept | 0.11286 | 1 | 0.11286 | 5.14 | 0.0248 |
| Slope | 0.0180172 | 1 | 0.0180172 | 0.82 | 0.3666 |
| Model | 31.3498 | 3 |  |  |  |

***P. gallicus* IT** Analysis of Variance

| Source | Sum of Squares | Df | Mean Square | F-Ratio | P-value |
| --- | --- | --- | --- | --- | --- |
| Covariates |  |  |  |  |  |
| T_a_ | 27.2551 | 1 | 27.2551 | 772.6 | 0.0 |
| Main effects |  |  |  |  |  |
| Model | 0.213028 | 1 | 0.213028 | 6.04 | 0.015 |
| Residuals | 5.75019 | 163 | 0.0352772 |  |  |
| Total (corr.) | 33.2799 | 165 |  |  |  |

Further ANOVA: Larvae vs. pupae

| Source | Sum of Squares | Df | Mean Square | F-Ratio | P-value |
| --- | --- | --- | --- | --- | --- |
| T_a_ | 27.3167 | 1 | 27.3167 | 769.7 | 0.0 |
| Intercept | 0.213028 | 1 | 0.213028 | 6 | 0.0154 |
| Slope | 0.000796765 | 1 | 0.000796765 | 0.02 | 0.8811 |
| Model | 27.5305 | 3 |  |  |  |

***P. biglumis* AT** Analysis of Variance

| Source | Sum of Squares | Df | Mean Square | F-Ratio | P-value |
| --- | --- | --- | --- | --- | --- |
| Covariates |  |  |  |  |  |
| T_a_ | 29.9114 | 1 | 29.9114 | 1241.86 | 0.0 |
| Main effects |  |  |  |  |  |
| Model | 0.0496175 | 1 | 0.0496175 | 2.06 | 0.1534 |
| Residuals | 3.39612 | 141 | 0.024086 |  |  |
| Total (corr.) | 33.3154 | 143 |  |  |  |

Further ANOVA: Larvae vs. pupae

| Source | Sum of Squares | Df | Mean Square | F-Ratio | P-value |
| --- | --- | --- | --- | --- | --- |
| T_a_ | 29.8696 | 1 | 29.8696 | 1232.42 | 0.0 |
| Intercept | 0.0496175 | 1 | 0.0496175 | 2.05 | 0.1547 |
| Slope | 0.00299342 | 1 | 0.00299342 | 0.12 | 0.7258 |
| Model | 29.9223 | 3 |  |  |  |

Table S6. Interspecific comparison of mass-specific metabolic rate of larvae and pupae. ANOVA was used to test for effects of temperature and species on metabolic rate. Further ANOVAs test for differences in intercept and slope in linear fits of species.

**Larvae** Analysis of Variance

| Source | Sum of Squares | Df | Mean Square | F-Ratio | P-value |
| --- | --- | --- | --- | --- | --- |
| Covariates |  |  |  |  |  |
| T_a_ | 43.9254 | 1 | 43.9254 | 1392.3 | 0.0 |
| Main effects |  |  |  |  |  |
| Model | 0.844066 | 2 | 0.422033 | 13.38 | 0.0 |
| Residuals | 7.13005 | 226 | 0.0315489 |  |  |
| Total (corr.) | 51.646 | 229 |  |  |  |

Further ANOVA: ***P. dominula* AT vs. *P. gallicus* IT**

| Source | Sum of Squares | Df | Mean Square | F-Ratio | P-value |
| --- | --- | --- | --- | --- | --- |
| T_a_ | 27.138 | 1 | 27.138 | 780.72 | 0.0 |
| Intercept | 0.676639 | 1 | 0.676639 | 19.47 | 0.0 |
| Slope | 0.0398149 | 1 | 0.0398149 | 1.15 | 0.2862 |
| Model | 27.8545 | 3 |  |  |  |

Further ANOVA: ***P. dominula* AT vs. *P. biglumis* AT**

| Source | Sum of Squares | Df | Mean Square | F-Ratio | P-value |
| --- | --- | --- | --- | --- | --- |
| T_a_ | 30.3516 | 1 | 30.3516 | 1073.59 | 0.0 |
| Intercept | 0.0151713 | 1 | 0.0151713 | 0.54 | 0.465 |
| Slope | 0.0264189 | 1 | 0.0264189 | 0.93 | 0.3353 |
| Model | 30.3932 | 3 |  |  |  |

Further ANOVA: ***P. gallicus* IT vs. *P. biglumis* AT**

| Source | Sum of Squares | Df | Mean Square | F-Ratio | P-value |
| --- | --- | --- | --- | --- | --- |
| T_a_ | 30.0666 | 1 | 30.0666 | 985.69 | 0.0 |
| Intercept | 0.538197 | 1 | 0.538197 | 17.64 | 0.0 |
| Slope | 0.137899 | 1 | 0.137899 | 4.52 | 0.035 |
| Model | 30.7427 | 3 |  |  |  |

**Pupae** Analysis of Variance

| Source | Sum of Squares | Df | Mean Square | F-Ratio | P-value |
| --- | --- | --- | --- | --- | --- |
| Covariates |  |  |  |  |  |
| T_a_ | 44.3375 | 1 | 44.3375 | 1833.39 | 0.0 |
| Main effects |  |  |  |  |  |
| Model | 0.00568093 | 2 | 0.00284046 | 0.12 | 0.8892 |
| Residuals | 5.58634 | 231 | 0.0241833 |  |  |
| Total (corr.) | 49.9346 | 234 |  |  |  |

Further ANOVA: ***P. dominula* AT vs. *P. gallicus* IT**

| Source | Sum of Squares | Df | Mean Square | F-Ratio | P-value |
| --- | --- | --- | --- | --- | --- |
| T_a_ | 31.0193 | 1 | 31.0193 | 1308.75 | 0.0 |
| Intercept | 0.0053018 | 1 | 0.0053018 | 0.22 | 0.6369 |
| Slope | 0.0995863 | 1 | 0.0995863 | 4.2 | 0.042 |
| Model | 31.1242 | 3 |  |  |  |

Further ANOVA: ***P. dominula* AT vs. *P. biglumis* AT**

| Source | Sum of Squares | Df | Mean Square | F-Ratio | P-value |
| --- | --- | --- | --- | --- | --- |
| T_a_ | 30.8755 | 1 | 30.8755 | 1706.66 | 0.0 |
| Intercept | 0.00026257 | 1 | 0.00026257 | 0.01 | 0.9043 |
| Slope | 0.000638967 | 1 | 0.000638967 | 0.04 | 0.8512 |
| Model | 30.8764 | 3 |  |  |  |

Further ANOVA: ***P. gallicus* IT vs. *P. biglumis* AT**

| Source | Sum of Squares | Df | Mean Square | F-Ratio | P-value |
| --- | --- | --- | --- | --- | --- |
| T_a_ | 26.8511 | 1 | 26.8511 | 894.31 | 0.0 |
| Intercept | 0.00286328 | 1 | 0.00286328 | 0.1 | 0.7579 |
| Slope | 0.0729108 | 1 | 0.0729108 | 2.43 | 0.1213 |
| Model | 26.9269 | 3 |  |  |  |

Table S7. Interspecific analysis of mass on metabolic rate. ANOVA was used to test for effects of temperature, species and mass on metabolic rate.

**Larvae** Multifactorial ANOVA

| Source | Sum of Squares | Df | Mean Square | F-Ratio | P-value |
| --- | --- | --- | --- | --- | --- |
| Covariable |  |  |  |  |  |
| mass (g) | 9.673 | 1 | 9.673 | 324.18 | 0.0 |
| Main effect |  |  |  |  |  |
| Species | 0.645019 | 2 | 0.32251 | 10.81 | 0.0 |
| T_a_ | 62.8319 | 4 | 15.708 | 526.44 | 0.0 |
| Residuals | 8.38451 | 281 | 0.0298381 |  |  |
| Total (corr.) | 81.9328 | 288 |  |  |  |

**Pupae** Multifactorial ANOVA

| Source | Sum of Squares | Df | Mean Square | F-Ratio | P-value |
| --- | --- | --- | --- | --- | --- |
| Covariable |  |  |  |  |  |
| mass (g) | 0.501233 | 1 | 0.501233 | 18.04 | 0.0 |
| Main effect |  |  |  |  |  |
| Species | 0.348923 | 2 | 0.174461 | 6.28 | 0.0021 |
| T_a_ | 63.5238 | 4 | 15.8809 | 571.59 | 0.0 |
| Residuals | 8.02947 | 289 | 0.0277836 |  |  |
| Total (corr.) | 78.3764 | 296 |  |  |  |

Table S8. Intraspecific analysis of “Nest” on metabolic rate. ANOVA was used to test for effects of different nests and temperature on metabolic rate.

***P. dominula* AT**

**Larvae** Multifactorial ANOVA

| Source | Sum of Squares | Df | Mean Square | F-Ratio | P-value |
| --- | --- | --- | --- | --- | --- |
| Covariable |  |  |  |  |  |
| T_a_ | 4.78104 | 1 | 4.78104 | 196.05 | 0 |
| Main effect |  |  |  |  |  |
| Nest | 0.731356 | 6 | 0.121893 | 5 | 0.0003 |
| Residuals | 1.48763 | 61 | 0.0243873 |  |  |
| Total (corr.) | 16.3307 | 68 |  |  |  |

**Pupae** Multifactorial ANOVA

| Source | Sum of Squares | Df | Mean Square | F-Ratio | P-value |
| --- | --- | --- | --- | --- | --- |
| Covariable |  |  |  |  |  |
| T_a_ | 7.90857 | 1 | 7.90857 | 800.69 | 0 |
| Main effect |  |  |  |  |  |
| Nest | 0.324058 | 6 | 0.0540097 | 5.47 | 0.0001 |
| Residuals | 0.770417 | 78 | 0.00987714 |  |  |
| Total (corr.) | 18.5764 | 85 |  |  |  |

***P. biglumis* AT**

**Larvae** Multifactorial ANOVA

| Source | Sum of Squares | Df | Mean Square | F-Ratio | P-value |
| --- | --- | --- | --- | --- | --- |
| Covariable |  |  |  |  |  |
| T_a_ | 4.83025 | 1 | 4.83025 | 318.47 | 0 |
| Main effect |  |  |  |  |  |
| Nest | 0.850911 | 6 | 0.141818 | 9.35 | 0 |
| Residuals | 1.04652 | 69 | 0.015167 |  |  |
| Total (corr.) | 18.4699 | 76 |  |  |  |

**Pupae** Multifactorial ANOVA

| Source | Sum of Squares | Df | Mean Square | F-Ratio | P-value |
| --- | --- | --- | --- | --- | --- |
| Covariable |  |  |  |  |  |
| T_a_ | 9 | 1 | 8.67273 | 512.42 | 0 |
| Main effect |  |  |  |  |  |
| Nest | 1 | 6 | 0.107722 | 6.36 | 0 |
| Residuals | 1 | 59 | 0.0169251 |  |  |
| Total (corr.) | 15 | 66 |  |  |  |

***P. gallicus* IT**

**Larvae** Multifactorial ANOVA

| Source | Sum of Squares | Df | Mean Square | F-Ratio | P-value |
| --- | --- | --- | --- | --- | --- |
| Covariable |  |  |  |  |  |
| T_a_ | 10.5003 | 1 | 10.5003 | 382.7 | 0 |
| Main effect |  |  |  |  |  |
| Nest | 0.795929 | 5 | 0.159186 | 5.8 | 0.0001 |
| Residuals | 2.11268 | 77 | 0.0274374 |  |  |
| Total (corr.) | 16.6156 | 83 |  |  |  |

**Pupae** Multifactorial ANOVA

| Source | Sum of Squares | Df | Mean Square | F-Ratio | P-value |
| --- | --- | --- | --- | --- | --- |
| Covariable |  |  |  |  |  |
| T_a_ | 11.016 | 1 | 11.016 | 336.08 | 0 |
| Main effect |  |  |  |  |  |
| Nest | 0.64628 | 8 | 0.080785 | 2.46 | 0.0203 |
| Residuals | 2.36005 | 72 | 0.0327785 |  |  |
| Total (corr.) | 16.8455 | 81 |  |  |  |

Fig. S1 Respirometry measurement chambers with (A) larvae and (B) pupae.

| A | B |
| --- | --- |
| 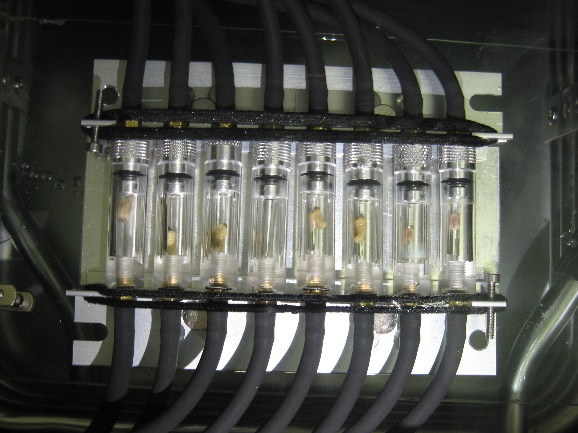 | 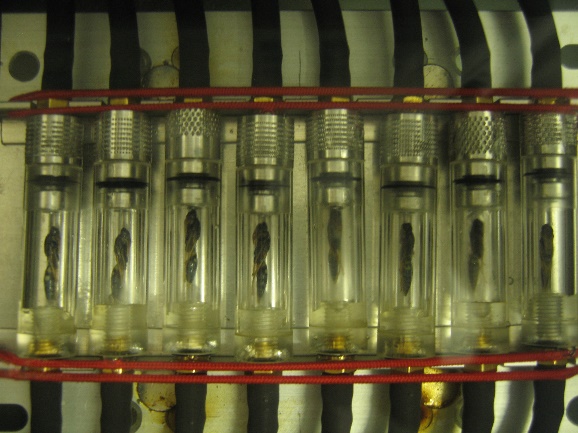 |

Fig. S2 Weight of investigated paper wasp larvae and pupae from Austria (*P. dominula* AT, *P. biglumis* AT) and Italy (*P. gallicus* IT). Box and whisker plots represent median weight with first and third quartiles; dots in plots are means (* significant difference p<0.05, Mann-Whitney test). The number above the plots represents the number of individuals.

Fig. S3 Nest temperatures of paper wasp larvae and pupae from Austria (*P. dominula* AT, *P. biglumis* AT) and Italy (*P. gallicus* IT) during a breeding season fom May to August in the years 2018 – 2023. Nest temperatures of *P. dominula* AT N1-8, *P. gallicus* IT N1-6 and *P. biglumis* AT N1-7 are modified data from Kovac et al. 2022. Climate data were recorded at six years (2018 – 2023), different breeding seasons were used for different species. Box and whisker plots represent median temperatures with first and third quartiles; dots in plots are means. The solid line in the background indicates the mean of all nests. The climate normal values (1981–2010) of the nearest weather stations in Austria (Graz, Schöckl ~20 km distance) and Italy (Florence, ~10–20 km) are indicated.

Fig. S4 Individual (A) and mass-specific (B) metabolic rate of paper wasp larvae and pupae from Austria (*P. dominula* AT, *P. biglumis* AT) and Italy (*P. gallicus* IT) in relation to ambient temperature (T_a_).

Fig. S5 Individual cumulative energetic expenditure of paper wasp larvae (A) and pupae (B) from Austria (*P. dominula* AT, *P. biglumis* AT) and Italy (*P. gallicus* IT) during a breeding season from May to August.

Fig. S6 Mass-specific cumulative energetic expenditure of paper wasp larvae (A) and pupae (B) from Austria (*P. dominula* AT, *P. biglumis* AT) and Italy (*P. gallicus* IT) during a breeding season from May to August.

Fig. S7 Representative raw respirometry traces for O_2_ and CO_2_ from a RQ experiment. Peaks represent O_2_ (blue) and CO_2_ (red) bursts of single respiratory measurement chambers during the flushing phase.


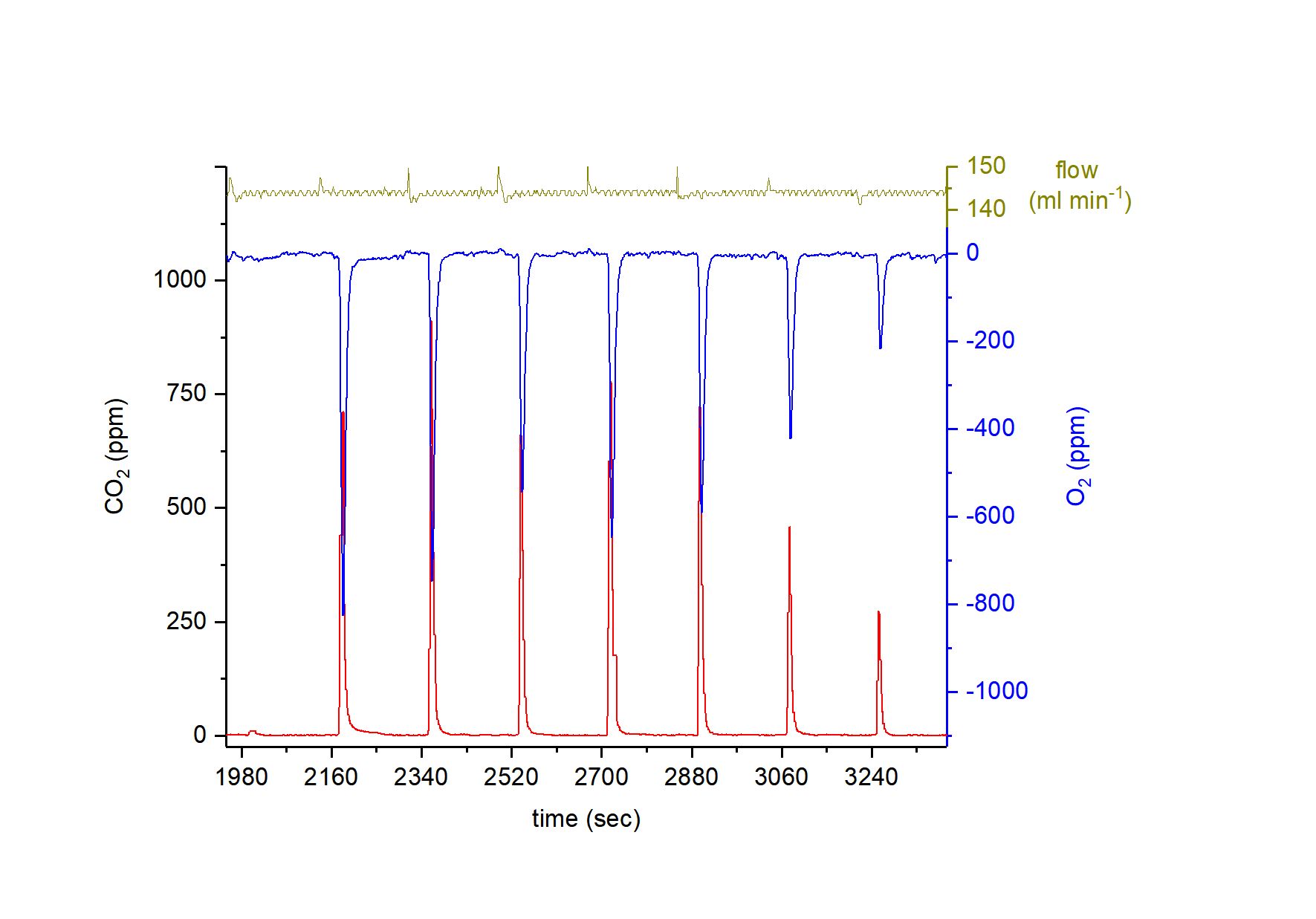

Supplement: Supplementary file 2 — Supplementary file2 (DOCX 3127 KB) [file 40_2025_1053_MOESM2_ESM.docx]
